# Molecular Detection of *Toxoplasma gondii* in Chickens and Ruminants: A Study From Kassena‐Nankana Districts, Ghana

**DOI:** 10.1002/puh2.70159

**Published:** 2025-10-25

**Authors:** Seth Offei Addo, Margaret Addo, Christopher Nii Laryea Tawiah‐Mensah, Emmanuel Kwame Amoako, Jennifer Nyamekye Yanney, Francisca Adai Torto, Richard Odoi‐Teye Malm, Stacy Amoah, Samuel K. Dadzie

**Affiliations:** ^1^ Parasitology Department, Noguchi Memorial Institute for Medical Research University of Ghana Accra Ghana; ^2^ Department of Medical Microbiology University of Ghana Medical School University of Ghana Accra Ghana; ^3^ Immunology Department, Noguchi Memorial Institute for Medical Research University of Ghana Accra Ghana

**Keywords:** Ghana, PCR, poultry, *Toxoplasma gondii*, zoonosis

## Abstract

*Toxoplasma gondii* is a zoonotic protozoan that poses serious threats to public health through contaminated animal products. The frequent interactions between inhabitants in the Kassena–Nankana districts and livestock increase the risk of zoonotic infections. However, there is limited information on the circulation of *T. gondii* within the districts. This study aimed to determine the prevalence of *T. gondii* infection in livestock species within the Kassena–Nankana districts using a PCR‐based diagnostic technique. Dry blood spots collected from February to December 2020 by convenience sampling were screened using PCR amplification of a 529 bp repetitive fragment specific to *T. gondii*. A total of 374 samples were collected from cattle (26.74%), chicken (26.20%), sheep (25.40%) and goats (21.66%). The overall prevalence of *T. gondii* was 13.9%, with all positives being from chicken samples. No infections were detected in the sampled cattle, sheep or goats. Free‐range chickens were more likely to be infected with *T. gondii* (odds ratios [OR] = 2, 95% confidence interval [CI] = 0.43–9.88, *p* = 0.38). Furthermore, it was observed that chicken from Namolo had a lower risk of infections (OR = 0.11, 95% CI: 0.02–0.43, *p* = 0.006). To reduce the dangers to public health, frequent education, improved poultry management and continuous surveillance within the districts are advised.

## Graphical abstract

• Chickens, especially free‐range chickens, play an essential role in the spread of *T. gondii*.

• To protect public health, there is a need for frequent education on the safe handling and proper cooking of poultry products.

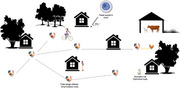

## 1 Introduction

The disease toxoplasmosis is caused by the zoonotic parasite *Toxoplasma gondii*, which infects warm‐blooded animals, including mammals and birds [1]. Human infection usually happens when a person consumes tissue cysts in raw or undercooked meat or consumes oocysts excreted by cats, which are the definitive hosts, or comes into contact with contaminated food and water sources [2]. Given these routes of transmission, livestock are an essential component of the zoonotic cycle, serving as intermediate hosts that allow the spread of the parasite to people through animal products. Thus, *T. gondii* infections in animals present serious health concerns to the general public, especially for those who eat raw or undercooked meat products [3]. It has been hypothesized that chickens, especially free‐range chickens, serve as important intermediate hosts for *T. gondii* and can be used as indicators of environmental contamination [4, 5]. Furthermore, chickens can play the role of sentinel animals in monitoring the risk of *T. gondii* infections in humans as well as examining the genetic diversity of the zoonotic parasite [6, 7, 8].

Veterinary specialists, farmers and abattoir workers are among the professionals who are known to be at risk for occupational exposure to *T. gondii* [9, 10]. Due to their frequent exposure to raw meat, animal blood and viscera, all of which may contain infectious stages of the parasite, these workers are at heightened risk [11]. As an example of the importance of occupational exposure, abattoir workers in some places have demonstrated increased seroprevalence of *T. gondii* antibodies compared to the general population [10, 12]. To lower the threat of human infections, the animal production and processing industries must improve their handling and hygiene procedures. An adequate strategy for treating *T. gondii* infections is provided by the One Health approach, which acknowledges the interdependence of environmental, animal and human health [13]. Targeting *T. gondii* in livestock by surveillance and control measures can lower the risk of its spread to humans, especially in areas where people often interact with animals and consume a lot of animal products. Integrated efforts combining environmental management, public health education and veterinary care are made easier by this multidisciplinary outlook.

In this regard, risk evaluation and management depend significantly on knowledge about *T. gondii* prevalence in animal populations. Some studies in Ghana have reported *T. gondii* infection or exposure in livestock [14, 15, 16]. However, there is limited molecular evidence of *T. gondii* in livestock, especially in Northern Ghana. In the Kassena–Nankana districts, inhabitants often interact closely with livestock through rearing or consumption, increasing the risk of infections. This study aimed to determine the prevalence of *T. gondii* in livestock from the Kassena–Nankana districts of Ghana using a sensitive PCR method to provide data that can inform public health and food safety interventions. For the detection of *T. gondii*, PCR is preferable to microscopy and serology because it provides more sensitivity and specificity for identifying the parasite's DNA, enabling diagnosis at the initial stages of infection or with minimal sample volumes [17]. One Health initiatives to reduce zoonotic transmission and enhance food safety will be informed by the identification of important animal reservoirs in the area.

## 2 Methods

**FIGURE 1:**
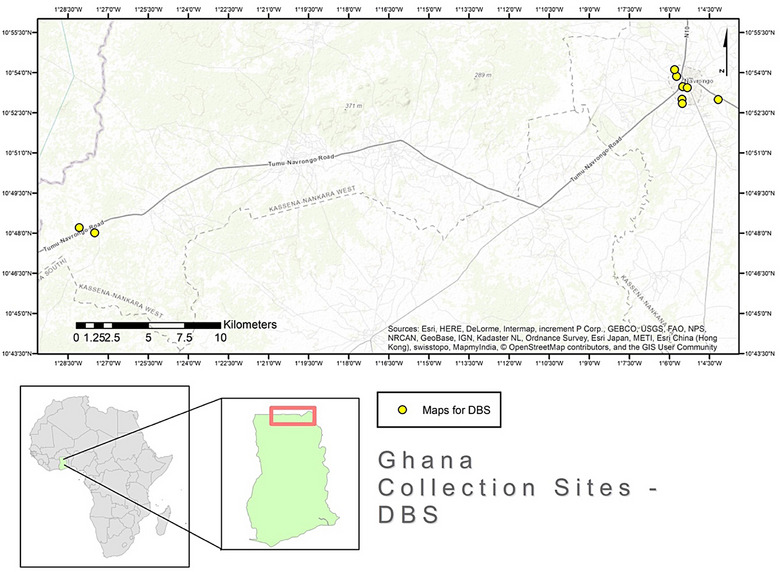
The map of Ghana indicating the sampling sites within the Kassena–Nankana districts for dry blood spot (DBS) collections.

This cross‐sectional study was conducted in nine locations within the Kassena–Nankana districts of Ghana (Figure 1). These included six sites within Navrongo, two sites in Nakong and a site in Pungu. From February to December 2020, convenience sampling was used to choose the animals based on availability at each location and verbal approval from each owner. With the aid of the animal owners, each animal was restrained, and samples were collected on the basis of the guidance of an attending veterinarian. The University of Ghana Institutional Animal Care and Use Committee (UG‐IACUC; UG‐IACUC 001/19‐20) granted ethical approval for this study.

### 2.1 Sample Collection

Employing Epi Info version 6, a minimum of 248 livestock were needed for this investigation. A 95% confidence level with a 5% error margin, a prevalence rate of 21.6% [18] and a population size of 5000 animals based on estimation by the local veterinarian were the assumptions used to determine the sample size. A total of 374 livestock, composed of cattle (100), chickens (98), sheep (95) and goats (81), were included in the study. Dry blood spot (DBS) samples were collected from cattle, sheep, goats and chickens. All the animals were physically healthy at the time of sampling. The samples were collected by an attending veterinarian following standardized protocols to ensure sample quality. With the cattle, sheep and goats, each animal was restrained, the facial vein disinfected and blood was drawn with the aid of a sterile animal lancet (Goldenrod Animal Lancet, Medipoint, NY, USA). Blood was also drawn from the wing vein of each chicken using the available lancet. In each case, the blood was spotted onto FTA cards (GE Whatman, Maidstone, Kent, UK), air‐dried, stored in collection bags containing silica gel and transported to the laboratory for storage at −80°C pending analysis.

### 2.2 DNA Extraction and Molecular Detection of *Toxoplasma Gondii* DNA

Using the Qiagen DNA Mini Kit (Qiagen Inc., Hilden, Germany), DNA was extracted from each DBS sample based on the manufacturer's instructions. The extracts were screened for *T. gondii* using primers TOX4 (CGCTGCAGGGAGGAAGACGAAAGTTG) and TOX5 (CGCTGCAGACACAGTGCATCTGGATT), which amplify the repetitive region of the 529 bp DNA fragment that repeats 200–300 times in the *T. gondii* genome [19]. The use of pre‐ and post‐PCR areas was employed to prevent contamination. Each PCR reaction consisted of 12.5 µL of GoTaq Hot Start Green Master Mix (2x), 6.5 µL of nuclease‐free water, 0.5 µM of each primer (forward and reverse) and 5 µL of the DNA template in a total volume of 25 µL. Negative (nuclease‐free water) and positive (*T. gondii* DNA) controls were included in each PCR reaction. The PCR was run using a Mastercycler X50‐PCR Thermocycler (Eppendorf, Germany) based on the cycling conditions: initial hold at 94°C for 7 min, followed by a second hold at 35 cycles at 94°C for a minute, 55°C for a minute and 72°C for a minute and finally the third hold at 72°C for 10 min. The reaction was held at 4°C. Subsequently, 5 µL of each PCR product was loaded on a 2% agarose gel, together with a 100 bp DNA ladder (New England BioLabs), and run at 110 V for 1 h 30 min. Upon completion of the run, the gel was observed in a Molecular Imager Gel Doc (Bio‐Rad).

### 2.3 Statistical Analysis

Descriptive statistics were used to characterize the animals which were sampled. This includes animal type, age, housing type, location of sampling and other characteristics. We then calculated *T. gondii* prevalence using the positivity rate and created confidence intervals (CIs) using the Clopper–Pearson exact approach. Pairwise Fisher's exact tests were performed when comparing the prevalence between species. To assess the associations of various locations and characteristics of the animal housing, we performed logistic regression using maximum likelihood. All analysis was conducted in R version 4.4.1.

## 3 Results

A total of 374 samples were collected from cattle (*n* = 100; 26.74%), chicken (*n* = 98; 26.20%), sheep (*n* = 95; 25.40%) and goats (*n* = 81; 21.66%). Most of the cattle were sampled from the cow market (48%), were females (51%) and were about 5 years old. The majority of the sampled chickens were sampled from Namolo (64%) and kept in deep litter housing type (64%). A further overview of the sampled animals is given in Table 1. The overall prevalence of *T. gondii* infection was 13.9%, with all positive cases found exclusively in chickens. The *T. gondii* infection rate in chicken was 53.06% (95% CI: 42.71%–63.22%). If statistically significant, odds ratios (OR) less than 1 indicate a protective effect against *T. gondii* infection, whereas OR values larger than or equal to 1 indicate increased risk. With regard to the housing types, the OR values did not confirm risk because they were not statistically significant, such as for free‐range housing (OR = 2.00, 95% CI 0.43–9.88, *p* = 0.38) (Table 2). The substantial protective association found in Namolo chickens (OR = 0.11, 95% CI 0.02–0.43, *p* = 0.006) suggests a lower probability of infection, most likely as a result of local husbandry or environmental practices (Table 3).

**TABLE 1:** Overview of sampled animals.

Characteristic	Cattle (*N* = 100)	Chicken (*N* = 98)	Goat (*N* = 81)	Sheep (*N* = 95)
Location, *n* (%)				
Abattoir	40 (40)	0 (0)	46 (57)	39 (41)
Chiefs Palace (Nogsenia)	0 (0)	15 (15)	20 (25)	29 (31)
Cow Market	48 (48)	0 (0)	0 (0)	0 (0)
Nakong (A)	2 (2.0)	0 (0)	0 (0)	0 (0)
Nakong (B)	8 (8.0)	0 (0)	0 (0)	0 (0)
Namolo	0 (0)	63 (64)	12 (15)	12 (13)
Nogsenia	0 (0)	5 (5.1)	0 (0)	0 (0)
Pungu	2 (2.0)	0 (0)	3 (3.7)	15 (16)
Tamgona (Notre Dame)	0 (0)	15 (15)	0 (0)	0 (0)
Animal sex, *n* (%)				
Female	51 (51)	98 (100)	71 (88)	72 (76)
Male	49 (49)	0 (0)	10 (12)	23 (24)
Age (years), median (IQR)	5.00 (3.00–7.00)	NA (NA–NA)	0.58 (0.50–0.80)	0.80 (0.58–1.20)
Unknown	0	98	0	0
Type of housing, *n* (%)				
Battery cages	0 (NA)	15 (15)	0 (NA)	0 (NA)
Deep litter	0 (NA)	63 (64)	0 (NA)	0 (NA)
Free range	0 (NA)	20 (20)	0 (NA)	0 (NA)
Unknown	100	0	81	95
Result, *n* (%)	0 (0)	52 (53)	0 (0)	0 (0)

*Note:* The location refers to the various sites where livestock samples were collected within the Kassena–Nankana districts, Ghana. ‘Abattoir’ is the main slaughterhouse; ‘Chiefs Palace Nogsenia’ is a traditional authority area; ‘Cow Market’ is the central livestock market; Nakong A and B are residential communities where livestock are reared; Namolo is a suburban area where many free‐range chickens were sampled; and Pungu and Tamgona Notre Dame are rural communities where livestock are kept.

Abbreviations: IQR, interquartile range; NA, not applicable.

**TABLE 2:** Association of housing type with *Toxoplasma gondii* among chickens.

	Counts			Odds	
Characteristic	Overall (*N* = 98)	Negative (*N* = 46)	Positive (*N* = 52)	OR (95% CI)	*p* value
Type of housing, *n* (%)					
Battery cages	15 (15)	5 (11)	10 (19)	—	
Deep litter	63 (64)	37 (80)	26 (50)	0.35 (0.10–1.11)	0.084
Free range	20 (20)	4 (8.7)	16 (31)	2.00 (0.43–9.88)	0.38

Abbreviations: CI, confidence interval; OR, odds ratio.

**TABLE 3:** Association of location with *Toxoplasma gondii* among chickens.

	Counts			Odds	
Characteristic	Overall (*N* = 98)	Negative (*N* = 46)	Positive (*N* = 52)	OR (95% CI)	*p* value
Location, *n* (%)					
Chiefs Palace (Nogsenia)	15 (15)	2 (4.3)	13 (25)	—	
Namolo	63 (64)	37 (80)	26 (50)	0.11 (0.02–0.43)	0.006
Nogsenia	5 (5.1)	2 (4.3)	3 (5.8)	0.23 (0.02–2.54)	0.22
Tamgona (Notre Dame)	15 (15)	5 (11)	10 (19)	0.31 (0.04–1.76)	0.21

Abbreviations: CI, confidence interval; OR, odds ratio.

## 4 Discussion

Animals raised for food are vital to public health because they act as reservoirs for human infections [20]. *T. gondii* was detected only in chicken samples in the Kassena–Nankana districts, indicating that poultry in Northern Ghana may be a major reservoir for this zoonotic parasite. The inability to detect the parasite DNA in the sampled cattle, sheep and goats could be due to some factors. Seroprevalence studies in ruminants confirm exposure and infection with *T. gondii*, despite occasional negative molecular detection [14, 15, 16, 21]. These findings highlight the fact that molecular negativity may indicate sampling and parasite dissemination constraints rather than ruling out infection [22]. Again, *T. gondii* infection in ruminants is often latent, with tissue cysts primarily seen in muscular and neural tissues. Depending on the type of tissue collected, the parasite burden is typically low and irregularly distributed, which can make PCR detection difficult. The amount of parasite DNA in blood or superficial tissue samples may be insufficient for detection [22]. Additionally, using dried blood spots can lower the yield of DNA, particularly in species with minimal parasitaemia. Consequently, negative PCR results emphasize the necessity for other sample methods, such as tissue or serological investigations, rather than ruling out infection.

The findings from this study can be compared to similar studies in Ghana and other tropical regions where backyard or free‐range poultry production is prevalent [5, 20, 23, 24]. As significant intermediate hosts, chickens get infected mainly by consuming oocysts released by felids that contaminate the environment, including feed, water and soil [1]. Within the Kassena–Nankana districts, the chickens are kept in poultry farms or allowed to roam free‐range. It was observed that both free‐range chickens and those kept in poultry farms recorded *T. gondii* infections. Free‐range chickens usually get infected with *T. gondii* when they feed on soil contaminated with cat or domesticated animal faeces [25]. Poor hygienic conditions in poultry farms can also facilitate the spread of *T. gondii* [26]. Better poultry management techniques that reduce exposure to *T. gondii* oocysts or localized environmental factors may be responsible for the notable protective effect seen in Namolo chickens. These results highlight the importance of taking local ecological and management factors into account when evaluating the threats of zoonotic diseases. On the other hand, non‐statistically significant OR values can indicate small sample sizes or variation in infection exposure, highlighting the need for more extensive or focused research to validate these correlations.

Chickens are crucial sentinels for environmental contamination with *T. gondii* oocysts because of their foraging behaviour, which raises the risk of exposure [6]. The spatial clustering of positive chicken samples, mostly in Navrongo, also points to localized environmental pollution or management strategies that promote parasite transmission. Implementing sentinel surveillance in free‐range chickens could allow early detection of environmental contamination hotspots, enabling timely public health responses. Although it makes sense to suggest chickens as sentinel animals for environmental *T. gondii* contamination due to their high infection prevalence and foraging habits, practical implementation must take into consideration difficulties like choosing representative sentinel populations, standardizing sampling procedures, guaranteeing laboratory capacity and securing cooperation from stakeholders across sectors. Before a large‐scale implementation, capacity‐building programs and pilot studies to evaluate viability and cost‐effectiveness are advised. Improved biosecurity measures, such as barring cats from accessing poultry feed and housing areas, enhancing sanitation and hygiene in poultry farms and, when practical, supporting controlled rearing systems over free‐range practices, should be the main focus of targeted changes in poultry husbandry to reduce zoonotic risk.

The frequent use of traditional slaughtering techniques that do not have strict hygienic regulations could aid in the spread of *T. gondii*. The threat may be increased by the sociocultural setting of the study location, where free‐range chickens are commonly raised and eaten [3]. In addition, occupational groups like poultry farmers, vendors and abattoir workers may be more susceptible to *T. gondii* due to direct contact with contaminated materials or infected animals, as has been shown in comparable environments [9, 10, 11]. Given previous reports of *T. gondii* exposure in pregnant women in Ghana [27, 28, 29, 30], our findings reinforce the need to control zoonotic sources in animals, especially poultry. Human exposure can be decreased by routinely monitoring livestock, particularly poultry and providing guides on safe meat handling and cooking practices, especially in areas such as Kassena Nankana, where local food systems and small‐scale livestock farming are major sources of income.

In terms of the One Health concept, the current molecular approach concentrates on detecting the parasite DNA in livestock; however, integrating human, animal and environmental surveillance is necessary for a comprehensive understanding of *T. gondii* transmission. To better characterize infection dynamics and risks, the One Health paradigm emphasizes interdisciplinary efforts incorporating molecular, serological and ecological data. Future studies that include tissue samples, human seroprevalence and environmental evaluations will offer a more complete picture that is necessary for successful control measures in the Kassena–Nankana districts.

Notwithstanding the significant findings, the study has some limitations. The convenience sampling and district‐limited coverage may bias prevalence estimates. To evaluate the risk of infections, there is a need for nationwide surveillance which takes into account longitudinal designs, tissue sampling, serological testing and molecular characterization of *T. gondii* strains. Furthermore, to measure the impact on public health, concurrent research evaluating human seroprevalence and risk behaviours is required.

## 5 Conclusion

The findings highlight chickens as a significant source of *T. gondii* infection within the Kassena–Nankana districts. Raising awareness among poultry farmers, merchants and consumers about the safe handling and proper cooking of poultry products should be the goal of locally specific educational initiatives. No infections were detected in the sampled ruminants. Future studies should broaden their geographic scope, incorporate tissue and serological samples, particularly in ruminants, and concurrently evaluate human seroprevalence and risk factors to fully describe the dynamics of zoonotic transmission and guide integrated control measures.

## Author Contributions

**Seth Offei Addo**: conceptualization, writing – original draft, investigation. **Margaret Addo**: investigation, writing – review and editing. **Christopher Nii Laryea Tawiah‐Mensah**: investigation, writing – review and editing. **Emmanuel Kwame Amoako**: investigation, writing – review and editing. **Jennifer Nyamekye Yanney**: investigation, writing – review and editing. **Francisca Adai Torto**: investigation, writing – review and editing. **Richard Odoi‐Teye Malm**: investigation, writing – review and editing. **Stacy Amoah**: investigation, writing – review and editing. **Samuel K. Dadzie**: investigation, writing – review and editing.

## Conflicts of Interest

The authors declare no conflicts of interest.

## Data Availability

All the data supporting this study are included in the article.
